# Automated measurement of the human corpus callosum using MRI

**DOI:** 10.3389/fninf.2012.00025

**Published:** 2012-09-12

**Authors:** Timothy J. Herron, Xiaojian Kang, David L. Woods

**Affiliations:** ^1^Human Cognitive Neurophysiology Laboratory, Research Service, US Veterans Affairs, Northern California Health Care SystemMartinez, CA, USA; ^2^Department of Neurology and Center for Neuroscience, University of CaliforniaDavis, CA, USA; ^3^Center for Mind and Brain, University of CaliforniaDavis, CA, USA

**Keywords:** morphometry, colossal commissure, gender, Alzheimer's disease, aging, splenium, genu, isthmus

## Abstract

The corpus callosum includes the majority of fibers that connect the two cortical hemispheres. Studies of cross-sectional callosal morphometry and area have revealed developmental, gender, and hemispheric differences in healthy populations and callosal deficits associated with neurodegenerative disease and brain injury. However, accurate quantification of the callosum using magnetic resonance imaging is complicated by intersubject variability in callosal size, shape, and location and often requires manual outlining of the callosum in order to achieve adequate performance. Here we describe an objective, fully automated protocol that utilizes voxel-based images to quantify the area and thickness both of the entire callosum and of different callosal compartments. We verify the method's accuracy, reliability, robustness, and multisite consistency and make comparisons with manual measurements using public brain-image databases. An analysis of age-related changes in the callosum showed increases in length and reductions in thickness and area with age. A comparison of older subjects with and without mild dementia revealed that reductions in anterior callosal area independently predicted poorer cognitive performance after factoring out Mini-Mental Status Examination scores and normalized whole brain volume. Open-source software implementing the algorithm is available at www.nitrc.org/projects/c8c8.

## Introduction

Neuroimaging of the corpus callosum has attracted great interest in both medical and neuroscience literature in the past few decades. Callosal changes due to brain atrophy have been characterized in Alzheimer's disease (Tomaiuolo et al., 2007; Di Paola et al., 2010; Frederiksen et al., 2011b), multiple sclerosis (Hasan et al., 2012b), and Huntington's disease (Di Paola et al., 2012) and callosal morphology has been related to symptom severity. Abnormalities in callosal morphology have also been reported in neuropsychiatric diseases including schizophrenia, bipolar disorder, and depression (Sun et al., 2009; Walterfang et al., 2009b; Bearden et al., 2011). In addition, developmental disorders (Paul, 2011) including Williams syndrome (Luders et al., 2007a; Sampaio et al., 2012), autism (Tepest et al., 2010), attention-deficit/hyperactivity disorder (Luders et al., 2009; Gilliam et al., 2011), and dyslexia (Hasan et al., 2012a) are associated with callosal abnormalities. The corpus callosum is also vulnerable to diffuse axonal injury and atrophy following traumatic brain injury (Maller et al., 2010). Finally, callosal changes are found during human development and aging (Sullivan et al., 2002; Hasan et al., 2008b; Luders et al., 2010b), with callosal morphology reflecting hemispheric asymmetries as well as gender differences (Bishop and Wahlsten, 1997; Luders et al., 2003, 2010a,b; Gurd et al., 2012).

The quantitative morphological analysis of the midsagittal corpus callosum is complicated by the interindividual variability of its size and shape (Thompson et al., 2003). Standard automated morphometric analyses that rely on standard whole-brain normalization of T1-weighted (T1W) images to a common stereotaxic template (Ashburner and Friston, 2000; Wang et al., 2009) often result in the imprecise alignment of the callosum because of variability in size and shape with respect to other brain structures (Dougherty et al., 2005; Chaim et al., 2007; Wang et al., 2009). More sophisticated deformation-based image normalization and coregistration techniques (Shen and Davatzikos, 2003; Huang et al., 2005; Sun et al., 2007; Tomaiuolo et al., 2007; Wang et al., 2009) have also been used to accurately map white matter (WM) into the same space. Strong deformation mapping into a unified space has the advantage that no callosal segmentation needs to be performed in individual images, only on the template or mean image. However, the robustness of deformation-based callosal analysis in multisite studies is not clear. Further, whole head coregistration based on a general-purpose optimization function lacks the flexibility that might be useful in mapping callosum subregions across subjects.

A variety of techniques have been introduced to accurately segment, align and measure the callosum (Bookstein, 2003; Thompson et al., 2003; Luders et al., 2007a; Sun et al., 2007; Wang et al., 2009; Adamson et al., 2011). However, most of these techniques are not completely automated and require manual intervention to outline the callosal boundaries (Ballmaier et al., 2008), correct WM segmentation (Walterfang et al., 2009a; Adamson et al., 2011), identify the tips of the callosum (Peters et al., 2002) or label other seed points (Niogi et al., 2007).

One valuable automated approach to automatically segmenting and measuring the callosum is provided by boundary-based callosal segmentation (Brejl and Sonka, 2000; Van Ginneken et al., 2002; Xu et al., 2007) and unified measurement protocols (Kubicki et al., 2008; Rotarska-Jagiela et al., 2008; Luders et al., 2010a; Frederiksen et al., 2011b). These algorithms generally require a training set of hand-segmented callosa to define a population-specific atlas of callosal templates. The templates can either be warped upon T1 callosal images to try and match new callosa, or can be used to define a shape or appearance model of the callosum. For example in the algorithm of Styner and colleagues (Styner et al., 2005; Kubicki et al., 2008), the training set is used to encode a boundary shape model parameterized by complex Fourier coefficients. The range of such coefficients then constrains the possible callosal boundaries that can be identified in a new subject. Best fit boundaries are aligned across subjects using a Procrustean alignment (Peterson et al., 2001; Bookstein, 2003) between evenly spaced boundary points determined over the Fourier-parameterized boundaries. Another sophisticated boundary- and atlas-based callosal segmentation and measurement system, developed by Stegmann and colleagues (Stegmann et al., 2004; Ryberg et al., 2007), was recently used in a multisite study of the effects of aging on the callosum (Ryberg et al., 2008; Frederiksen et al., 2011a). Boundary-based methods using atlases have the advantage of superior performance in automatically segmenting the callosum from the fornix and pericallosal artery because permissible callosal shapes can be strongly constrained by the atlas or its derived shape models. The main disadvantage of boundary-based methods is the necessity of developing a population-specific atlas defining permissible callosal shapes and subsequent potential inaccuracies in quantifying callosa with unusual shapes that may occur in other patient populations.

Rule-based callosal segmentation algorithms are capable of automatically segmenting the callosum without manually defined templates (Lee et al., 2000). For example, Schönmeyer and colleagues (Schönmeyer et al., 2007; Rotarska-Jagiela et al., 2008) recently developed a rule-based image processing algorithm that uses relatively homogenous image intensity values to define image objects that are then used for callosal segmentation. Such image objects must be present in certain absolute locations of the image and have particular positions with respect to each other for the rules to properly segment the callosum. The rules include explicit procedures for detaching the fornix. However, Schönmeyer's algorithm has not been tested on a large database of images from different sources and does not provide measures of callosal thickness or overall length. Rule-based callosal measurement methods have several advantages. First, they are computationally simple and hence can be executed rapidly. Second, because they do not depend on parameterizing the upper and lower callosal boundaries they are less impacted by boundary errors (Lee et al., 2000) than template-based approaches. Third, rule-based algorithms are less vulnerable to measurement errors in subjects with unusual callosal shapes or divided into multiple clusters. The main disadvantage of rule-based algorithms is that they contain only relatively crude implicit shape information, and hence can be vulnerable to segmentation errors, particularly in failing to accurately segment the callosum from the fornix and pericallosal arteries.

In the current study, we introduce a novel fast, fully automated rule-based technique that does not require manual callosal segmentation. We introduce methods for (1) automatically isolating and parcellating the callosum, (2) defining standard locations along the length of the midsagittal corpus callosum, and (3) estimating callosal thickness centered on those standard locations as well as quantifying areas within geometrically defined callosal compartments (Witelson, 1989; Hofer and Frahm, 2006). Then we validate the performance of these automated procedures, collectively named C8, in four separate tests using publicly available structural brain imaging datasets (Marcus et al., 2007; Biswal et al., 2010): (1) We compare the results of our method with the results obtained using manual callosal segmentation. (2) We evaluate the robustness of the method to variations in image preprocessing procedures and to variations in image resolution. (3) We compare the test–retest reliability of our method with manual segmented images in subjects who underwent repeated scans. And finally, (4) we test the influences of different scanners on callosal morphology. These tests establish that C8 provides accurate callosal measurements regardless of image preprocessing or image resolution and that these measures show high test–retest reliability and comparability across different scanners.

We then use C8 to characterize callosal changes in normal aging and mild dementia. First, we analyze healthy control data from subjects of different ages to examine the influences of age, sex, and intracranial volume (ICV) on callosal size and morphology. The results show significant changes with both age and ICV. Second, we compare callosal size and shape in older, mildly demented adults with matched controls and find that the size of anterior callosal compartments adds predictive information about cognitive outcome.

## Materials and methods

Our overall approach is to first use standard algorithms to produce whole-brain WM segmentations that are then used for callosal quantification: standard spatial affine normalization algorithms applied to the T1W image are used to warp the WM segmentations into Montreal Neurological Institute (MNI) space. Specialized callosal cluster detection algorithms are then used to define the cross-sectional midline portion of the callosum along its full extent. Finally, voxel-based measurements are taken by summing the segmentation values in 2D (areas) and along line segments in 1D (thicknesses) in MNI space and inverting the normalization to obtain original image space values. It is worth noting that although we identify a callosum boundary during the clustering step, we do not parameterize the boundary curves in order to define superior and inferior callosal surfaces for use in normalization or for making measurements (Sun et al., 2007; Kubicki et al., 2008; Ryberg et al., 2008; Wang et al., 2009; Luders et al., 2010b; Adamson et al., 2011). In this sense C8 is a voxel-based method as opposed to a surface-based method (Clarkson et al., 2011).

### Thickness definition and rationale

**Figure 1:**
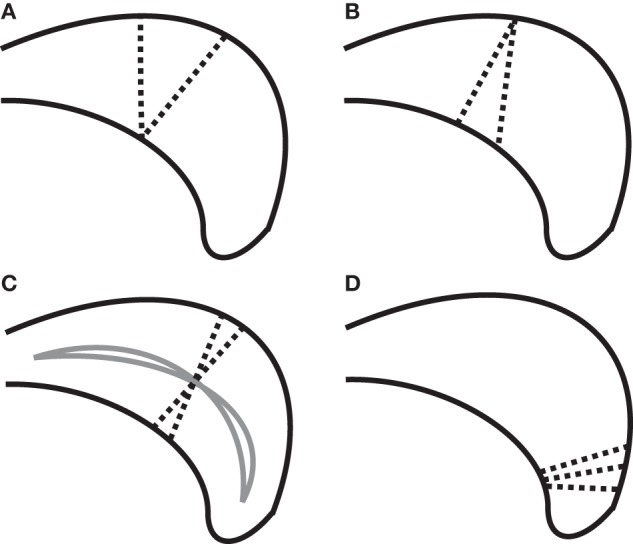
**Complexities in defining thickness (using dotted line segments) shown on a cartoon posterior callosum. (A)** Problem with definition by minimal traversal distance (vertical line is shorter). **(B)** Line segments defining thickness cannot always be perpendicular to both boundaries simultaneously. **(C)** Sensitivity of thickness to median anchor point (solid lines) when perpendicularity to the median line is required. **(D)** High boundary curvature on only one surface causes fans of thickness-defining lines that nearly intersect; complicating attempts to define thickness using uniformly spaced grid lines.

Defining callosal thickness is complicated by the shape variability of the callosum. For example, one early definition of callosal thickness used the length of line segments stretching between two sets of corresponding, evenly spaced anchor points on the superior and inferior callosal boundaries (Peters et al., 2002; Luders et al., 2003). However, beyond the key requirement to accurately define callosal endpoints that separate the inferior and superior boundaries, this definition results in inflated thickness values if the superior and inferior boundaries have different curvatures and lengths that introduce offsets in corresponding anchor points. Figure 1 shows several possible defining properties of thickness and the potential problems each property has due to varying callosal shape. For example, it is problematic to require that thickness defining line segments be perpendicular with respect to either the boundaries (Figure 1B), because boundaries may not be parallel in corresponding locations; or with respect to an interior line (Figure 1C), because interior path location or curvature errors can also inflate thickness values.

We chose here to use the minimum traversal distance to define thickness: namely, the length of the shortest line segment across the callosum that intersects a given interior anchor point on a median line defined along the length of the callosum (see section “Measuring Thickness, Area, and Length” for details). Interior anchor points, as opposed to boundary anchor points, are used in order to minimize the incidence of inappropriately small values (Figure 1A). An important aspect of the minimal traversal distance is that is it a fully local definition: the thickness estimates in one location are not dependent upon the shape of the corpus callosum in distant areas.

**Figure 2:**
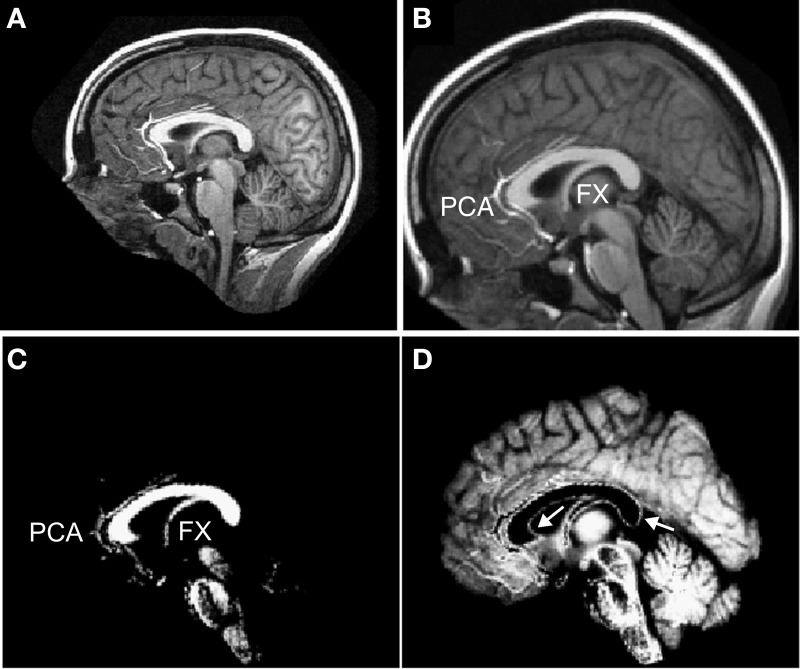
**Preprocessing of T1 images.** Each T1-weighted MR image **(A)** was normalized into MNI space **(B)** and segmented into white matter **(C)** and gray matter **(D)**. Labels: FX = fornix and PCA = peri-callosal arteries. Arrows indicate incorrectly segmented WM voxels at the callosal boundary in the GM partition.

A challenge in making a fully automated algorithm to isolate and measure the corpus callosum is to make the method robust to segmentation failures. In particular, it is often challenging to algorithmically disconnect the callosum from the fornix (Lee et al., 2000; Schönmeyer et al., 2007) and from the pericallosal artery (Figure 2C). Although we implemented several methods to remove the fornix and pericallosal arteries from the callosum WM cluster (see section “Callosal Identification”), we were not successful in removing them in all cases. However, because our method provides local measures, thickness misestimations are limited to those localities where the callosum is connected to another structure like the fornix. Further, our minimum traversal distance definition of thickness also will tend to avoid using fornix WM or blood vessel voxels that fail to be properly excised from the callosum cluster because the shortest line segment across the callosum will generally avoid callosal attachments.

### Brain images

For the evaluation of our method as well as for sample applications, we used images taken from two public T1W image databases. First, we used the OASIS high-resolution anatomical image database (Marcus et al., 2007)^1^. There are a total of 416 right-handed subjects in the database, including 152 young normals (age 18–39; 20 with repeated scans), 98 demented older subjects (age 60–96), 100 cognitively normal older subjects (age 60–94), and 66 middle-aged controls (age 40–59). All subjects underwent three or four anatomical T1W sagittal scans (1.5T MP-RAGE, voxel size 1.0 mm × 1.25 mm × 1.0 mm with TR/TE/FA = 9.7 ms/4.0 ms/10°) that were averaged together to create one high resolution T1W image for analysis. Every subject's age and sex was recorded. For older subjects, education, socioeconomic status, the Mini-Mental State Examination (MMSE) score and the clinical dementia rating (CDR) (Morris, 1993) were also obtained. Finally, a set of machine-generated callosal segmentations for all 316 healthy controls from the Automatic Registration Toolbox (ART) project^2^ were hand-corrected for segmentation errors and served as a performance reference.

Second, T1W whole-brain image data from 1231 subjects from the 1000 Functional Connectomes Project (FCP) database were also analyzed (Biswal et al., 2010)^3^. The subset of this database that we analyzed originated from 25 different sites (see Supplementary Table S1 for details) and included age and sex as covariates for all subjects: 54% female, age range 13–85 (71% age 18–29), and ~95% right-handed. Each utilized subject's dataset included one high-resolution T1W image and had in plane resolution of 1.2 mm or less. We excluded 24 anatomical images because either normalization to MNI space or whole head tissue segmentation failed using site-specific scripts.

### Image preprocessing

T1W images were segmented into gray matter (GM), WM, and cerebrospinal fluid (CSF) compartments using SPM5^4^, which assigns probabilities to each voxel that reflect the likelihood that the voxel belongs to GM, WM, or CSF (Figure 2). SPM5 tissue segmentation uses a clustering analysis that starts with an apriori template of tissue locations (warped from MNI space) and iteratively solves for mixtures of tissue types present in each voxel (Ashburner and Friston, 1997). In order to examine the influence of different automated segmentation algorithms, the OASIS images from young normal subjects were also segmented with SPM8's unified segmentation (Ashburner and Friston, 2005), with FreeSurfer's^5^ WM segmentation algorithm (Dale et al., 1999), and with the expectation-maximization algorithm EMS (Van Leemput et al., 1999)^6^. SPM8's unified segmentation is similar in overall approach to SPM5's cluster-based design but incorporates nonlinear registration of prior probability maps during classification giving it somewhat improved performance (Salvado et al., 2007; Klauschen et al., 2009; De Bresser et al., 2011). FreeSurfer's segmentation algorithm specializes in labeling WM for the purpose of generating cortical surfaces by combining sophisticated spatial and histogram intensity normalization with specialized boundary detection algorithms. Overall, FreeSurfer's segmentations have excellent quality but tend to slightly over assign voxels to WM when compared to expert segmentations (Klauschen et al., 2009). The EMS algorithm uses an iterative expectation-maximization algorithm to predict tissue types based on image intensity, starting from a T1W image template, while also imposing Markov random field structure to enhance neighborhood agreement on tissue type. EMS has been demonstrated to achieve nearly the same performance as SPM5 but with different technical characteristics; in particular EMS WM segmentations tend to assign too few brain voxels to WM (Salvado et al., 2007).

For all of the above segmentation algorithms, default parameters were used except for SPM5 and SPM8. For those, it was found that reducing the brain voxel sample spacing parameter to 2 mm (default: 3 mm) substantially improved the accuracy of the resulting segmentation. Unfortunately, the tissue segmentations performed by all tested image preprocessing packages contain voxels that, because of partial voluming effects, are miscategorized as GM (particularly evident on the inferior callosal surface in Figure 2D). Thus, we expected that our callosal measurements would underestimate the thickness of the callosum in comparison with measurements based on manual callosal segmentation.

Finally, the T1W images were also normalized to MNI space following a 12 parameter affine transformation using SPM5 (Figure 2B). The segmented images were then normalized to MNI space and resliced to isotropic 1 mm^3^ voxels (or isotropic 8 mm^3^ voxels for some analyses) using trilinear interpolation.

### Morphometric analysis of the corpus callosum

C8's fully automated analysis procedure was used to identify and measure the corpus callosum by analyzing the normalized WM segmentations generated as described above. The callosum on the mid-sagittal plane and on each of two adjacent parasagittal slices (at x = ±1 mm in MNI space) were isolated and analyzed separately. Thus, the analysis procedures described below were applied to all three para-midsagittal slices, and median values of the final derived quantities were used in order to increase the robustness of the overall procedure. Multiple callosal slice analysis has been used previously (Rotarska-Jagiela et al., 2008) and improves performance because the callosal shape profile changes only gradually away from the midline (Luders et al., 2006a).

#### Callosal identification

Within each slice, a bounding box was defined in MNI space using a probabilistic map of the mid-sagittal callosum based on post-mortem brains (Burgel et al., 2006). C8 initially searched for callosal clusters within this box. Using seed points dropped down from a callosum's superior surface plus a WM cluster growing procedure, contiguous sets of WM voxels were selected on the sagittal plane and the boundaries of the callosal clusters were identified using a fixed WM segmentation value threshold. Note that this procedure allows for the possibility of generating multiple clusters of midsagittal callosal voxels that could occur in cases of disease, malformation, or the rare instance of a normal subject having a very thin isthmus that appears to separate the callosum into two parts.

We used four techniques to reduce the incidence of apparent fornix or callosal artery attachments. First, prior to identifying callosal clusters, any segmented WM voxel that could not be placed on some locally linear WM path within 45 degrees of the medial-to-lateral direction (i.e., “Y” direction in MNI space) was removed. This reflects the fact that callosal fibers are expected to primarily traverse the callosum in a mediolateral direction, a fact used previously by others in analyzing diffusion images to isolate and measure the callosum (Hasan et al., 2008b). Second, the aforementioned analysis of three para-midsagittal slices often resulted in only one of those callosal clusters containing fornix WM. In such cases, the faulty measurements from that single slice were significantly discounted in the final estimate by using median values of all estimates. Third, the use of a fixed WM segmentation threshold to define the interior of the callosal clusters often helped assign the fornix and other non-callosal structures to separate clusters that could then be ignored by restricting analysis to the largest (and longest along the anterior–posterior axis) cluster. Fourth, after obtaining the putative callosal WM cluster, we erased any WM cluster branch (a WM cluster segment separated by non-WM voxels) that was inferior to the main body of the cluster along the callosal mid body within a specified MNI range. The use of these four techniques generally limited fornix contamination to only a small part of the fornix remaining attached to the callosum.

#### Defining standard callosal partitions

**Figure 3:**
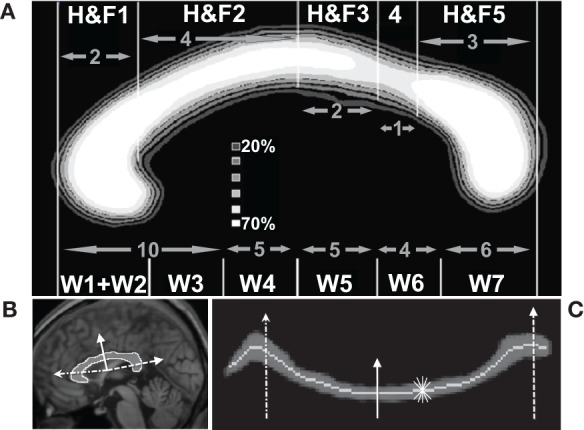
**Quantification of callosal area and thickness. (A)** Average callosal structure of 152 young subjects from the OASIS database, averaged in MNI space. Each subject's callosum was subdivided into five compartments along the anterior–posterior axis using geometric ratios following Hofer and Frahm (H&F, top of panel **A**) (Hofer and Frahm, 2006) and divided into six compartments following Witelson (W, bottom of panel **A**) (Witelson, 1989). **(B)** Callosal boundaries were defined with reference to a series of radial lines (three shown) emanating from a centroid. **(C)** Radial lines intersecting the callosum were oriented vertically. This unwrapped the callosum to define a median line and measure thickness. The same three lines intersecting the callosum in **(B)** are shown. The light gray line shows the median location of WM probabilities (dark gray) considered vertically. Callosal thickness was computed at each point using the shortest line segment connecting the superior and inferior surfaces through that point (five shown, short thin white).

The geometric partitioning schemes proposed by Hofer and Frahm (Hofer and Frahm, 2006) and Witelson (Witelson, 1989) were used to segment the CC into topographic compartments. The maximum extent of the CC along its anterior–posterior axis was identified, and parcellated into five or six compartments based on geometric ratios (Figure 3). The Hofer and Frahm parcellation incorporates a representation of five subregions of the human callosum based on diffusion imaging fiber tractography (Hofer and Frahm, 2006). The cortical parcellation is as follows: Compartment 1 to prefrontal cortex, Compartment 2 to premotor and supplementary motor cortex, Compartment 3 to primary motor cortex, Compartment 4 to primary sensory cortex, and Compartment 5 to parietal, temporal, and occipital cortices. This geometric parcellation is similar to the scheme introduced by Witelson, based on non-human primate data (Witelson, 1989), that has been widely used to assess callosal pathology (Thompson et al., 2003).

#### Measuring thickness, area, and length

The thickness at each point along the length of the corpus callosum was computed as the minimum distance between the probabilistic boundaries of the callosum measured with line segments cutting across the callosum that intersected points on a median line (defined below) in the sagittal plane. Sums of automatically generated segmentation probabilities are commonly used to compute brain volumes, e.g., as in Kruggel (2006), and the summing technique allowed us to compute thicknesses and areas while avoiding the difficult task of defining callosal boundaries to subvoxel accuracy. Similarly, defining corpus callosum thickness as the minimum distance computed using variously angled short line segments passing through one point avoids the difficult problem of defining the correct perpendicular line with respect to the callosal boundaries or to the median itself. A similar technique has been successfully used to produce reliable cortical thickness measurements (Fischl and Dale, 2000) although it may produce slight underestimates of thickness due to image noise or mismatched boundary shapes (Figure 1). The median callosal line was determined over the entire length of the callosum (Figure 3) by using a series of radial lines at 1.65° intervals emanating from a centroid located halfway between the most anterior and posterior extents of the callosum and along the inferior–superior axis at the most inferior extent of the splenium. Our centroid is slightly superior to the Hampel centroid often used to divide the callosal into partitions radially (Hampel et al., 1998). A median callosal point was defined along each radial line as the median WM location using WM segmentation probabilities squared as median weights within an 11.55° neighborhood. Thicknesses were measured through each of these median points and then interpolated to obtain values at 50 equal-angle spaced points from the anterior tip to the posterior tip of the callosum.

Mean thickness (in mm) and total callosal area (in mm^2^) were then computed for each of the five callosal compartments. All measurements of thickness and area, performed within standard MNI space, were transformed back to native anatomical space by inverting the affine spatial normalization transformation computed for each individual brain. Thus, C8 provides both MNI space values and original anatomical space values—each has their use depending upon the application (Jäncke and Steinmetz, 2003; Luders et al., 2006b). In this manuscript we will use native space estimates unless otherwise indicated. Finally, the 50 anchor-point standardized median line allows us to compute an estimate of the total internal callosal length in MNI space by summing the lengths separating adjacent median line anchor points and inverting the affine transform to provide native space length estimates.

### Method accuracy, reliability, robustness

C8's accuracy and reliability were first evaluated by making callosal measurements on structural MRI data from the 152 normal control subjects contained in the publicly available OASIS high-resolution anatomical image database (Marcus et al., 2007). Visually inspecting the callosal segmentations isolated by C8 suggested that when fornix or pericallosal artery adhesions were evident on multiple para-midsagittal slices they were generally limited to a few voxels and would therefore have little effect on regional thickness and area measurements.

We performed several comparisons to evaluate the reliability of our morphometric procedures. We analyzed CC areas within the Witelson partitions (see Figure 3A) for each of the 152 healthy young OASIS controls (OASIS-152) and compared them with the results of previous studies which used expert manual CC delineation on similar datasets from young, healthy right-handed subjects (Jäncke et al., 1997; Bermudez and Zatorre, 2001; Luders et al., 2003, 2006a; John et al., 2008). We also evaluated the effects of image resolution by comparing C8 measurements performed on three resolutions of affine normalized segmentation images—0.125, 1, and 8 mm^3^ isotropic voxels. Finally, we compared the C8 callosal estimates with those computed using expert-corrected callosal segmentations from the ART database. ANOVA statistical comparisons and power estimates, using non-central F distribution models, were computed using CLEAVE^7^.

The OASIS database also contains repeated anatomical scans for a subset of 20 young, normal subjects (OASIS-20) that were used to estimate the scan-to-scan reliability of C8 measurements. We further manually delineated the callosa within these 40 images as an additional test of C8's accuracy. The manual segmentation was done on anonymized OASIS-20 T1W images (40 total) affine-normalized to MNI space by a trained member of our laboratory not otherwise affiliated with this study. In addition to correlations between repeated scans, we also computed fractional Dice coefficients (Crum et al., 2006) to measure the overlap between automated and manually corrected segmentations.

A third set of tests checked the robustness of C8 to different segmentation algorithms. We compared results from the OASIS-152 dataset within the Hofer and Frahm (H&F) partitions using the SPM5, SPM8, FreeSurfer, and EMS segmentation algorithms. Mean values of area and thickness are reported within three H&F partitions as well as correlations between these values across segmentation types.

A fourth set of tests aimed to validate the consistency of the area and thickness measurements. We used the estimated distances in native space between adjacent median line thickness measurement locations combined with local thickness measures to produce local area measurements that should sum to the total callosal area measurement. Thus, this test verified how well area values (2D sums of segmentation probabilities) compared with thickness values generated by searching for minimal 1D sums of (interpolated) segmentation values across the callosum.

Finally, we evaluated the reliability of C8 across image sets by analyzing T1 image data taken from 14 different MR scanners within the FCP image database (Biswal et al., 2010) that contain comparable young, healthy subjects of both sexes (group mean age <36 y.o., sex ratio between 1:2 and 2:1). First, we compared regional mean callosal thickness values across the groups. Second, we performed ANOVAs and multivariate linear regressions using the MatLab Statistics toolbox^8^ in order to measure variation in callosum areas due to scanner/group differences vs. those due to age, sex, overall brain volume, and image quality. Image quality was parameterized in two ways: first by voxel size (Y: anterior/posterior and Z: superior/inferior) in the sagittal plane and second by computing the image entropy (−*∫*_*v*∈*ICV*_*v* · ln *v* · *dv*) over all intracranial voxel (ICV) intensity values *v*. Intracranial voxels are defined as voxels primarily segmented as being in WM, GM, or CSF in the preprocessing stage. Image entropy measures the overall lack of distinctness in the image as reflected in the histogram of image values: we normalize the maximum entropy value, given by a uniform distribution of values, to 1.

### Applications

#### Age-related changes in the callosum

We first analyzed C8 callosum measurements from all FCP subjects (25 sites, 1231 subjects) using linear regression to evaluate the effects of age, sex, total brain volumes estimated from the accompanying segmentation images, and image quality. We computed quadratic regression curves for effects of age on both callosal thickness and median length. We also repeated the analysis using all 316 normal controls from the OASIS dataset for both automatically segmented callosa and expert corrected segmentations. We then computed the full Spearman correlation matrix for the above values in order to view the regional callosal area and thickness covariates of age, sex, and brain volume. In these statistical analyses, we discarded four obvious outlying measurements of older subjects from each of the FCP and OASIS datasets. The outlier data were due to poor image contrast, unusual spatial fluctuations in anatomical image values, or excessive WM hypointensities that interfered with SPM5's whole brain segmentation's clustering algorithm.

#### Callosal alterations in dementia

Our second analysis compared CC values of 98 mild Alzheimer-related dementia cases from the OASIS dataset (age 76.7 ± 7.1 years, 58 female) to those with 98 age-matched older controls (age 75.9 ± 9.0 years, 72 female) to evaluate if callosal measurements contain information helpful to classifying mild dementia. This data were previously used to show (Marcus et al., 2007) that normalized whole brain volume (nWBV), the fraction of brain matter contained within the total ICV, was a useful predictor of mild dementia as defined by the CDR score (Morris, 1993). Here, we measured the callosum areas within the five Hofer and Frahm partitions to see if they provide additional separation power to distinguish normal aging from mild dementia. We used subject demographic information of age, sex, and education (five-point scale) along with the MMSE score (Folstein et al., 1975), nWBV, total ICV, and the five H&F partition areas within an ordinal logistic regression, using the Design library in R ver. 2.13^9^, to predict the CDR score: 0 for controls; or 0.5 (very mild) and 1.0 (mild) for dementia patients.

## Results

### Method accuracy, reliability, robustness

Table 1 shows the measurements of corpus callosum area obtained using C8 fully automatically or when using expert corrected segmentations. The results are comparable with earlier reports using manual callosal tracing as shown in Table 1, albeit with the expected small underestimation of CC area of the automatically segmented C8 data. Similarly, thickness measures produced by the minimum traversal distance definition used above were slightly smaller than those reported with manual callosal delineation. For example, the average thickness in H&F compartments 2 and 3 for the 152 young, normals from the OASIS database was 5.3 ± 0.7 mm. Mean thicknesses for the callosal body computed in prior studies using manual tracing (not using a minimal traversal distance definition) averaged 6–7 mm for two groups of young controls (Luders et al., 2007a,b) reported by Luders and colleagues, and averaged 7.2 ± 1.9 mm in young controls reported by Raine and colleagues (Raine et al., 2003). However, a previous study using semi-automated methods applied to voxel intensity-based WM segmentations produced mid-body callosal thicknesses of approximately 6.0 mm (Walterfang et al., 2009a,b), closer to our own.

**Table 1:** **Corpus callosum area measurements (mean and standard deviations) for callosal Witelson (W) compartments (see Figure 3A) obtained from the OASIS-152 anatomical image database (Marcus et al., 2007) using the present method**.

**Study**	**Voxel size**	**W1 + W2 + W3 mm^2^**	**W4 + W5 mm^2^**	**W6 mm^2^**	**W7 mm^2^**
C8 OASIS-152 fully automated analysis (*n* = 152, age: 23.2 ± 4.2)	2 mm	240 ± 38	128 ± 25	49 ± 13	163 ± 30
		1 mm	243 ± 36	130 ± 20	50 ± 12	166 ± 23
		0.5 mm	241 ± 35	130 ± 21	51 ± 12	165 ± 23
OASIS-152 manually corrected segmentations		260 ± 36	140 ± 20	56 ± 13	185 ± 26
Jäncke et al., 1997; *n* = 54, age 27.8 ± 5.2		274 ± 34	162 ± 24	66 ± 12	186 ± 31
Bermudez and Zatorre, 2001; *n* = 136, age: 24.6 ± 4.8		294 ± 34	169 ± 12	67 ± 14	190 ± 27
Luders et al., 2003; *n* = 30, age: 23.3 ± 3.9		254 ± 38	149 ± 11	57 ± 13	191 ± 29
Luders et al., 2006a; *n* = 60, age: 23.9 ± 4.7		235 ± 39	117 ± 14	44 ± 11	177 ± 34
John et al., 2008; *n* = 23, age = 30.1 ± 5.1		260 ± 37	151 ± 25	252 ± 29	
C8 FCP database analysis [*n* = 969, age: 23.2 ± 3.9 (18–35)]		231 ± 35	125 ± 22	50 ± 13	159 ± 25

Results using 2 mm, 1 mm, and 0.5 cubed WM segmentations are given in Rows 1–3. Row 4 contains the results from the C8 analysis of the manually corrected ART segmentations as processed in non-normalized (but AC-PC aligned) image space. For comparison, results reported in five earlier studies using hand-segmentation are presented as well as results automatically computed with our method for the lesser image quality FCP database (bottom row).

**Figure 4:**
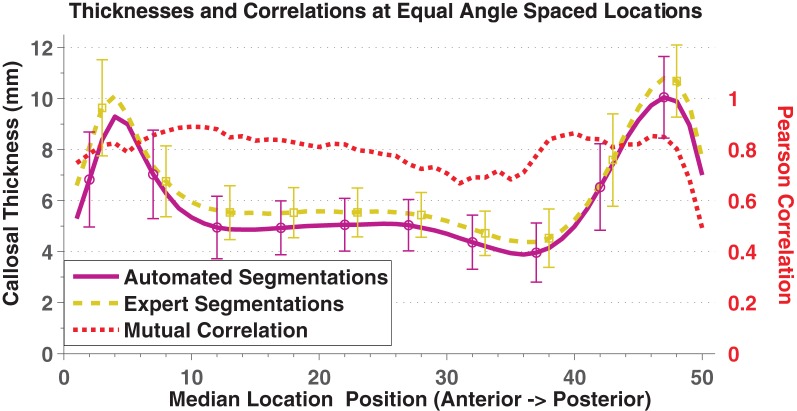
**Mean and standard deviation (error bars) thickness at 50 equal angle spaced locations using automated segmentation (purple solid) and expert-corrected segmentations (gold dashed).** The red dotted line indicates the Pearson correlations between the two values at each location.

In direct comparisons of the OASIS-152 subjects using fully automated segmentations vs. expert-corrected segmentations, thickness correlations were fairly high across most of the median callosal locations (Figure 4). Similarly, Pearson correlations between the automated and expert segmentations within Hofer and Frahm partitions were 0.87, 0.86, 0.68, 0.89, and 0.95, from anterior to posterior (and 0.92 for total area). Thus, only the thin H&F3 callosal compartment adjacent to the fornix and the tip of the splenium had relatively reduced correlations.

Repeated scan reliability of the mean area and thickness measurements are reported in Table 2, using the OASIS-20 subjects who underwent two scanning sessions. Intersession variability (~5%) was markedly less than the intersubject variability (>15%) shown in Table 1. We have found no previous callosum intersession variability numbers for comparison, however comparable *volume* variability for healthy controls's shapewise-similar subcortical structures of the caudate and hippocampus range from about 1% to 3% (Jovicich et al., 2009).

**Table 2:** **Mean absolute % differences in thickness and area within the five Hofer and Frahm (H&F) compartments across two imaging sessions for 20 subjects who underwent repeated scanning in the OASIS-20 database**.

	**Voxel size (mm)**	**H&F1 (%)**	**H&F2 (%)**	**H&F3 (%)**	**H&F4 (%)**	**H&F5 (%)**
Thickness variation	1	4.1	2.1	4.3	4.2	3.8
	2	4.0	2.7	6.1	6.4	4.8
Area variation	1	5.0	5.1	5.8	6.4	1.5
	2	6.0	6.5	6.7	4.7	2.7

Odd rows show values computed using segmentations with 1 mm side voxels, while 8 mm^3^ isotropic voxels were used for the even rows' data.

**Figure 5:**
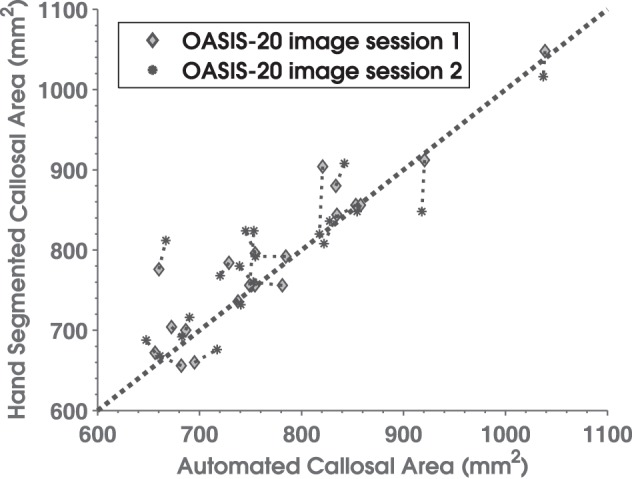
**Scatterplot comparing manually delineated corpus callosum total cross-sectional areas (y-axis) with C8 area estimates (x-axis) for 20 normal subjects in the OASIS database who underwent repeated scans.** Diamonds are from image session 1 and asterisks are for session 2. Thin dotted lines connect results for the same subject's two sessions, while the thick dotted line is the area diagonal.

Manually delineated total CC areas for the OASIS-20 images correlated strongly with C8 estimates as shown in Figure 5. C8's automated method produced a consistent and accurate (*r* = 0.90, Pearson correlation overall) estimate of callosal areas. This correlation to the gold-standard of hand segmenting the T1W images (as well as the 0.92 derived previously for the OASIS-152 data) compares favorably to a Pearson correlation of 0.80 obtained by Hasan and colleagues using a semi-automated DTI-based method (Hasan et al., 2008a). However, C8's correlation with manual segmentation was less than most inter-rater correlations of total area measurements under repeated hand-segmenting of single images by different experts, which have correlation that often exceed 0.95 (Bermudez and Zatorre, 2001; Dorion et al., 2002; Horton et al., 2004; Wang et al., 2006; Ballmaier et al., 2008; Tepest et al., 2010). The 40 automated callosal segmentations and hand segmented callosa overlap each other with a mean fractional Dice coefficient of 0.89 (0.04 SD), which is not as high as a previously reported value (Schönmeyer et al., 2007) of 0.97 (but where Schönmeyer threw out one of 50 images as an outlier). Higher overlap values with C8 would not be expected because SPM5's whole-brain segmentation algorithm assigns some of the callosal boundary to the discarded GM partition (Figure 2).

Table 3 shows the effects of different segmentation preprocessing algorithms on C8. Correlations between the area and thickness values produced by processing different WM segmentations were high with thickness showing generally higher cross-segmentation correlations across the callosum. Finally, processing more than three adjacent mid-sagittal slices also made only minor differences in mean values of area or thickness (see Supplementary Table S3).

**Table 3:** **Pearson correlation coefficients for area (upper triangular, bold) and thickness (lower triangular, italic) values between C8 computations applied to the OASIS-152 dataset using four different segmentation preprocessing methods (see the Image Preprocessing section and Supplemental Table S2) within three of the H&F partitions (see Figure 3)**.

	**H&F1**				**H&F3**				**H&F5**				
	**SPM5**	**EMS**	**FS**	**SPM8**	**SPM5**	**EMS**	**FS**	**SPM8**	**SPM5**	**EMS**	**FS**	**SPM8**	
**SPM5**		**0.95**	**0.83**	**0.91**		**0.97**	**0.92**	**0.92**		**0.99**	**0.97**	**0.99**	**SPM5**
**EMS**	*0.95*		**0.84**	**0.88**	*0.97*		**0.93**	**0.93**	*0.98*		**0.97**	**0.99**	**EMS**
**FS**	*0.93*	*0.90*		**0.80**	*0.97*	*0.94*		**0.91**	*0.97*	*0.96*		**0.97**	**FS**
**SPM8**	*0.96*	*0.92*	*0.93*		*0.96*	*0.94*	*0.97*		*0.96*	*0.95*	*0.94*		**SPM8**

Next, we checked the consistency of thickness measurements with respect to area measurements by computing local thickness values multiplied by local median line distance measurements. We compared the sums of all such locally computed area values with the total callosal area measured simply by summing the segmentation values. We found that the local thickness times median length values had a mean area deficit of only −0.9% (SD 1.6%). Thus, assuming that accurate callosal median length values were used, thickness measurements showed only a slight negative bias. However there is a slight correlation (Pearson *r* = −0.17) between the bias and the size of the callosum, with larger CCs having larger discrepancies (see Supplementary Figure S4).

**Figure 6:**
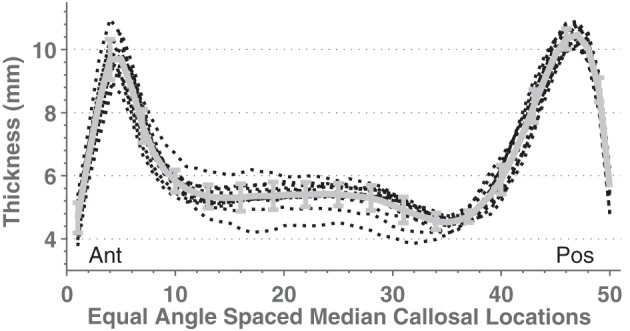
**Mean mid-sagittal callosal thicknesses from 14 different scanners (black dotted lines) with young (age means 21–33 years), mixed gender, healthy subjects taken from the FCP database (see Supplemental Table S1).** The thick gray line shows the mean and standard deviation.

In order to test C8 on multisite data, we compared callosal estimates across different scanners by using 14 image sets containing young, normal, gender-balanced subjects within the FCP database (864 subjects total, mean age 24.2, SD 6.0). We obtained regional group mean thickness values at 50 equal-angle-spaced locations along the CC shown in Figure 6. Mean thickness values were similar across sites although the mid-body values had higher relative variance.

ANOVAs performed on mean callosal thickness, median length, and total area all showed statistically significant omnibus differences across the 14 groups: *F*_(13, 850)_ = 3.4, 12.3, and 7.1, respectively (all *p* < 0.001, Greenhouse-Geisser corrected). However, the effect sizes of the group differences are fairly small: intergroup standard deviations are 3.5, 2.9, and 5.5% for thickness, median length and area compared to mean intersubject standard deviations of 11.6, 7.3, and 13.5%, respectively. These site differences largely reflected demographic differences in subject populations and image quality. When the potentially relevant covariates of age, sex, brain volume (from WM and GM segmentations), voxel size, image entropy, and indicator variables for group membership were included within a regression of callosum measurements (Table 4), no sites's values were statistically different (Bonferroni corrected) from those of the most average group. In all cases, however, image voxel dimensions and image entropy differed between groups [entropy ANOVA: *F*_(13, 850)_ = 11.6, *p* < 0.0001]. Thus, two sources of group variation were accounted for otherwise with image entropy being the most significant. Recomputing the regressions after discarding the group indicator variables resulted in small but significant Z voxel dimensions for mean callosal thickness (*t*_844_ = 3.9) and Y voxel dimensions for callosal length (*t*_844_ = 4.1).

**Table 4:** **Regression equation coefficients and *t*-values computed from young, normal FCP data from 14 groups for overall thickness, median length, and area (columns)**.

	**Callosum thickness**		**Callosum length**		**Callosum area**	
	**Coeff. (mm)**	***t*_(844)_**	**Coeff. (mm)**	***t*_(844)_**	**Coeff. (mm^2^)**	***t*_(844)_**
Age (year)	0.001	0.2	0.113	2.8^*^	0.759	1.4
Female	−0.121	2.1	1.619	3.6^*^	−1.924	0.3
Brain vol. (cm^3^)	−0.003	1.0	0.027	14.3^*^	0.192	7.7^*^
Normed image entropy [0–1]	30.77	9.7^*^	−40.01	1.6	2552	8.0^*^
Y voxel size (mm)	4.277	0.4	−27.15	0.3	−150.4	0.1
Z voxel size (mm)	−6.286	0.5	41.89	0.5	89.7	0.1
Group indicators	0 groups *t*_(844)_ > 2.8^*^		0 groups *t*_(844)_ > 2.8^*^		0 groups *t*_(844)_ > 2.8^*^	

Dependent variables (rows) were age, sex, brain volume, intracranial normalized image entropy (from 0 to 1; 1 = uniform image intensity distribution), image voxel sizes, and group indicator variables. Each column contains the estimated regression coefficient (left) along with the t-value (right).

* p < 0.05/19.

The results in Table 1 and Figure 4 show that the variance of C8 measurements of Witelson partition areas fell within the range of variance obtained in studies using manual callosal segmentation. We next compared the statistical power of C8 and manual analysis in two ANOVAs: one comparing male and female callosal OASIS-152 measurements and another comparing OASIS-152 subjects, divided into two groups that showed above and below median ICVs. The gender comparison yielded insignificant differences with either type of segmentation (e.g., males had 2.0% greater total area, *p* > 0.2). However, as suggested by Table 4, subjects with larger ICVs had increased callosal length. This effect was detected with similar statistical power using C8's automated segmentations [by 5.7%, *F*_(1, 150)_ = 33.2, *p* < 0.0001; power: 50 subjects give a 90% chance of *p* < 0.05] and manually corrected segmentations [by 5.4%, *F*_(1, 150)_ = 29.0, *p* < 0.0001; power: 57 subjects give a 90% chance of *p* < 0.05]. Mean callosal thickness also increased with higher ICV, both with C8 [by 4.7%, *F*_(1, 150)_ = 7.5, *p* < 0.01; power: 242 subjects give a 90% chance of *p* < 0.05] and manually corrected segmentations [by 4.4%, *F*_(1, 150)_ = 6.8, *p* < 0.01; power: 268 subjects give a 90% chance of *p* < 0.05].

### Applications

#### Age-related changes in the callosum

Age effects on total callosal mean thickness, and length were examined with repeated measures ANOVAs using the OASIS data from 152 young normals and 95 older normal subjects (age 60+) with both C8 and expert-corrected segmentations as factors. Here, we found consistent effects of age on callosal thickness [younger greater by 19.9%, *F*_(1, 245)_ = 155.1, *p* < 0.0001] and segmentation type [as expected, manual segmentation showed greater thickness by 8.4%, *F*_(1, 245)_ = 621.1, *p* < 0.0001]. There was also a significant interaction of age and measurement type [older subjects' segmentation difference increased to 13.1%, *F*_(1, 245)_ = 53.2, *p* < 0.0001], presumably reflecting age-related differences in WM segmentation by SPM5. Nevertheless, the statistical power of detecting age effects was similar for C8 and manual segmentation: younger subjects had larger thickness in both C8 segmentations [by 23.7%, *F*_(1, 245)_ = 181.8, *p* < 0.0001; power: 16 subjects give a 90% chance of *p* < 0.05] and manually corrected segmentations [by 16.6%, *F*_(1, 245)_ = 116.3, *p* < 0.0001; power: 23 subjects give a 90% chance of *p* < 0.05]. Callosal length showed an age effect [older greater by 6.4%, *F*_(1, 245)_ = 42.3, *p* < 0.0001] but, as expected, no significant difference between C8 and manually corrected segmentation [*F*_(1, 245)_ = 0.7, NS] or interaction between segmentation type and age [*F*_(1, 245)_ = 0.6, NS]. Age-related *increases* in callosal length were also detected with similar statistical power using C8 [by 6.0%, *F*_(1, 245)_ = 39.4, *p* < 0.0001; power: 68 subjects give a 90% chance of *p* < 0.05] and manually corrected segmentations [by 6.2%, *F*_(1, 245)_ = 43.8, *p* < 0.0001; power: 61 subjects give a 90% chance of *p* < 0.05].

**Figure 7:**
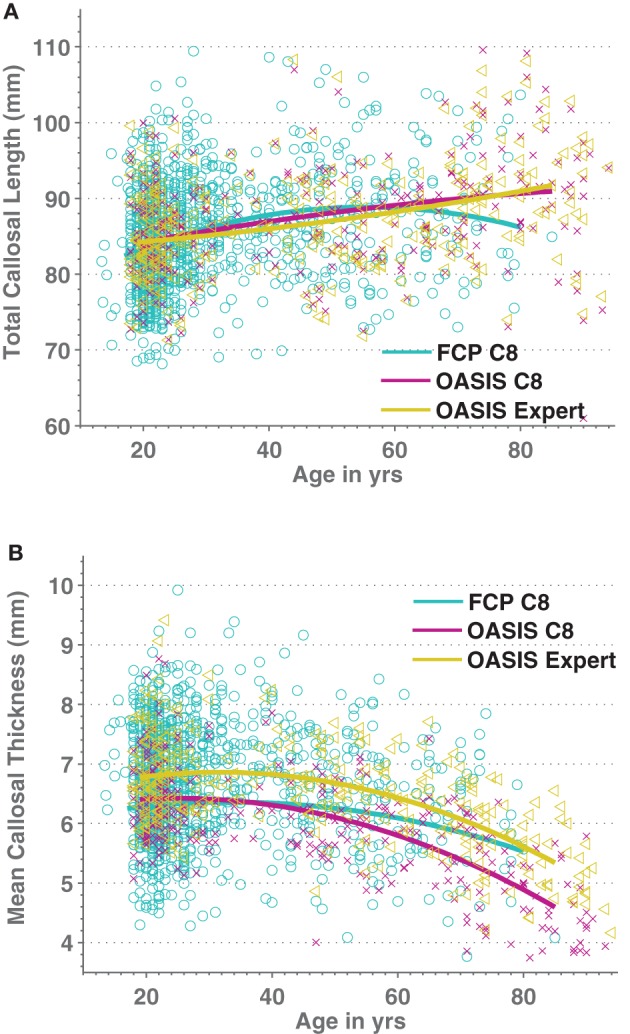
**Scatterplots of Age vs. medial callosal length (A) and Age vs. mean callosal thickness (B) for 1227 FCP database images processed by C8 (cyan/circles) as well as for both automated (purple/crosses) and expert-corrected (gold/triangles) segmentations of 316 OASIS control subjects.** Estimated quadratic age regression lines are included.

We then added 66 middle-aged OASIS subjects to the above older and younger subject groups and regressed both callosal thickness and median length against demographic, overall brain volume, and image quality data while ignoring indicator variables for the different scanner groups (we found only two groups in each of the resulting regression that differed significantly from the most representative group). We modeled age as a quadratic function to account for possible non-linear age-related differences. Figure 7 shows the resulting quadratic regression curves associated with age for callosal thickness and length (callosal area is shown in Supplemental Figure S5). Callosal thickness showed a gradual decline throughout adulthood. In contrast, callosal length generally increased with age, though the FCP data shows a drop after age 60. The callosal-length regression curves for C8 and manual segmentations were largely superimposed whereas the mean thickness curves were separated but parallel. The regression equation parameters with respect to age in the two OASIS regression pairs in Table 5 are highly consistent.

**Table 5:** **Regression equation coefficients computed using healthy subject FCP data from 25 groups and OASIS healthy control data, for both automatic and expert-corrected segmentations, and for overall thickness and median length (columns)**.

	**Mean CC thickness (mm)**			**Median CC length (mm)**		
**Dataset**	**FCP**	**OASIS automated**	**OASIS manual**	**FCP**	**OASIS automated**	**OASIS manual**
Age (year)	0.003	−0.020^*^	−0.014^*^	0.229^*^	0.114^*^	0.105^*^
Age^2^	−3.8e−4^*^	−5.1e−4^*^	−5.3e−4^*^	−4.4e−3^*^	−7.2e−4	6.4e−4
Female	−0.018	0.098	0.079	0.035	0.264	0.287
Brain vol. (cm^3^)	1.1e−3^*^	1.3e−3^*^	1.2e−3^*^	1.9e−2^*^	2.1e−2^*^	2.1e−2^*^
Normed image entropy [0–1]	18.23^*^	N/A	N/A	5.650	N/A	N/A
*Z* voxel size (mm)	−0.085^∧^	N/A	N/A	1.288	N/A	N/A

Dependent variables (rows) were age, age^2^, sex, brain volume, and for the FCP data the intracranial normalized image entropy (from 0 to 1; 1 = uniform image intensity distribution) and z image voxel size (superior-inferior direction). Each column contains the estimated regression coefficient. Age dependencies are graphed in Figure 7 and Supplemental Figure S5.

∧ *p < 0.05*,

* p < 0.005 (Bonferroni corrected).

Sex did not produce significant effects on callosal morphology whereas the effects of total brain volume were highly significant. Finally, in the FCP data the image quality measure of intracranial image entropy and slice thickness, voxel size only correlated with callosal thickness, implying that thickness measurements increased both with less distinct images and with smaller voxels.

**Figure 8:**
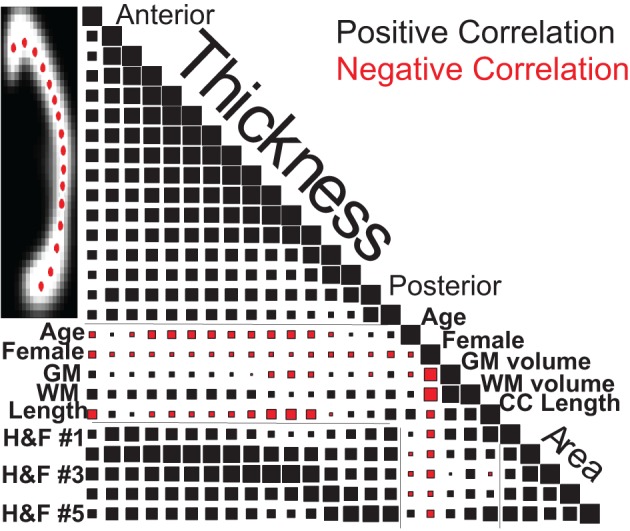
**Spearman correlation values for callosal thicknesses at 16 locations (Ant: anterior, Pos: posterior), regional callosal areas (within 5 H&F partitions: see Figure 3), and callosal length along with estimated GM and WM volumes, age and gender for the 1231 FCP subjects.** Square areas are proportional to correlation values, both positive (black) and negative (red), with correlations of 1.0 on the diagonal for scale.

Figure 8 graphically represents the Spearman correlation matrix for the variables used above plus H&F area and regional thickness measurements from 16 callosal locations. Regional callosal thickness and area correlated strongly and length correlated positively with area. However, surprisingly, overall callosal length correlated negatively with anterior and mid-body callosal thickness, most notably in the genu and around the isthmus. Females had smaller callosal measurements overall, particularly in the anterior callosum, but these correlations were smaller than the correlations of sex with brain volume for both WM and GM. The age-related reduction in callosal thickness shown in Figure 7 appears to be concentrated in the mid-body of the callosum while the age-related increase in callosal length is confirmed in Figure 8. Finally, whole-brain WM volume is positively correlated with callosal size and area regional measurements whereas whole-brain GM volume is positively correlated with callosal area, excluding the mid-body, but was not strongly correlated with callosal thickness. Similar correlations were obtained using the OASIS dataset (Supplemental Figure S6).

#### Callosal alterations in dementia

There were significant callosal area differences between normal adult images and those of patients diagnosed with abnormal Cognitive Dementia Ratings (CDRs) in the OASIS dataset. CDR-related area reductions within Hofer and Frahm (H&F) callosal partitions were, from anterior to posterior, 7.1, 12.3, 3.2, 9.1, and 8.9%, with H&F partitions 1, 2, 4, and 5 showing significant (*p* < 0.05) reductions in patients with mild dementia. In order to evaluate potential clinical utility of callosal measures in predicting the incidence of mild dementia, we applied a multivariate ordinal logistic regression to H&F partition areas along with the relevant available demographics indicators plus normalized brain volume, total ICV, and the score on the mini-mental status exam (MMSE). The results are shown in Table 6 which show that the MMSE is clearly the strongest predictor of the CDR, followed by total ICV and the anterior callosal compartment (H&F1) which both trend toward significance. When only the top three significant factors were reanalyzed; MMSE, eTIV, and H&F1 area; both eTIV and H&F1 area reached significance (Wald Z of 3.07 and −2.88, respectively, both *p* < 0.005). Thus, C8's estimates of anterior callosum area may be independently predictive of cognitive decline even accounting for cognitive screening and overall cranial size.

**Table 6:** **Ordinal logistic regression parameter values and significance values (*Z* and *p*-values) for predicting the Clinical Dementia Rating (CDR) scores in 191 older subjects within the OASIS dataset**.

	**Log odds parameter**	**Wald *Z* score**	***p*-value**
MMSE	−0.504	−7.39	0.0000
eTIV	2.641	1.99	0.0464
H&F1 Area	−0.018	−1.78	0.0752
H&F2 Area	−0.017	−1.38	0.1674
Age	−0.031	−1.17	0.2426
Education	−0.154	−1.17	0.2425
nWBV	−5.565	−0.87	0.3852
H&F3 Area	0.022	0.82	0.4117
Female	−0.266	−0.68	0.4963
H&F5 Area	0.004	0.60	0.5515
H&F4 Area	0.001	0.02	0.9811

Independent predictor values include demographic, brain volume (eTIV) and density (nWBV), neurocognitive MMSE score, and regional callosal area (H&F) values.

## Discussion

The methodology presented here permits a fully automated quantification of corpus callosum size and area despite variable callosal morphology (Thompson et al., 2003; Wang et al., 2009) that is adequate for large group studies. The C8 algorithm provides reliable measurements of callosal area, thickness, and median length that correlate strongly with hand-segmented callosal values. Furthermore, the C8 method is robust to differences in preprocessing algorithms used for whole-head tissue segmentation as well as to differences in image resolution. The statistical power obtained using C8 callosal measurement estimates is similar to that obtained using expert-segmented images because the *random* error added by C8 (e.g., Figure 4 error bars) is only a small fraction of natural inter-subject callosal variation.

### Limitations

We note five main limitations of the method. (1) The C8 algorithm is dependent upon the WM tissue segmentation preprocessing that is used—any improvements in whole brain segmentation algorithms, especially in the context of more challenging clinical populations, would increase the reliability and usefulness of C8. Even under ideal conditions, most image intensity clustering tissue segmentation algorithms result in the misclassification of some voxels on the callosal boundaries due to partial voluming (Figure 2D). As a result, in comparison with manual segmentation, C8 underestimates callosal area and thickness (but not length) though reliably across repeated sessions. (2) In addition, reductions in WM image intensity values, e.g., as in the older OASIS subjects, can cause slight but significant additional reductions in callosal area compared with manual segmentation. Thus, C8 might not be the best choice for quantifying callosal area in patients with demyelinating diseases. (3) The definition of callosal thickness as the minimum traversal distance, using summed segmentation values to define distance, slightly underestimates callosal thickness in comparison with alternative definitions of thickness, thus complicating the values C8 produces in comparison to previous literature. (4) An additional problem is faced in patient populations with substantial parenchymal loss (e.g., stroke victims) because affine normalization into MNI space may be compromised. However, simple rigid-body transformation of the mid-sagittal plane into approximately MNI space coordinates, e.g., as with the manually corrected OASIS segmentations above, allows C8 to process the realigned WM tissue. (5) Finally, shape-based callosal segmentation methods, such the one embodied in the open-source CCSeg software (Vachet et al., 2012)^10^, likely separate the fornix from the callosum more reliably, potentially reducing random measurement error.

### Applications

#### Age-related changes in the callosum

We used C8 to analyze the influence of age, sex, ICV, and image quality on a large, multi-site cohort of moderate-quality T1W images of healthy subjects from the FCP dataset as well as for the high-quality images from OASIS control subjects. Callosal area was reduced with age in these cross-sectional datasets, consistent with some previous reports (Sullivan et al., 2002; Suganthy et al., 2003; Hasan et al., 2008a,b) but not all (Lee et al., 2009). The quadratic aging function that we obtained for callosal total area predicts a loss of area of 0.87%/year (OASIS) and 0.78%/year (FCP) at age 75 (Supplemental Figure S5), similar to the 0.9%/year loss at age 75 found in a recent longitudinal study (Sullivan et al., 2002). Also, we found that callosal area appeared to peak in middle age, as in previous reports (Sullivan et al., 2002; Suganthy et al., 2003; Hasan et al., 2008a,b). This might reflect the fact that WM myelination increases with age and might even peak during those same years in many studies (Ardekani et al., 2007; Hasan et al., 2008a,b; Grieve et al., 2011) but is not a universal finding (Silver et al., 1997; Armstrong et al., 2004; Benedetti et al., 2006; Sullivan et al., 2010). Higher myelination might well lead to brighter T1 image values and thus greater overall WM segmentation volumes, most notably by increasing T1 values in the partially volumed boundary locations. However, the fact that it was callosal length rather than callosal thickness that drove the quadratic aging function to peak (Figure 7; Supplemental Figure S5) makes this explanation unlikely: one would expect greater changes in thickness than length with changes in WM segmentation. Finally, it has been noted previously that the callosum can increase significantly in length during healthy adult aging (Suganthy et al., 2003; Takeda et al., 2003; Gupta et al., 2008).

As in most previous studies, we found that callosal area and thickness varied only weakly with gender (Driesen and Raz, 1995; Bishop and Wahlsten, 1997). We found that males had slightly larger callosal area in native space with the reverse being true in MNI space (Smith, 2005; Luders et al., 2006b). In addition, we found that GM volume correlated moderately positively with callosal length but only weakly with callosal thickness. Finally, there were small negative correlations between callosal length and regional thicknesses.

#### Callosal alterations in dementia

We also found that alterations in callosal morphology could aid in predicting mild dementia after brain volume, basic neurocognitive tests, and demographics were taken into account. Although straightforward group comparisons show that posterior callosal areas are reduced in mildly demented populations as expected (Wang et al., 2006; Frederiksen et al., 2011b), presumably reflecting the parietal and temporal atrophy that occurs in Alzheimer's disease (Hampel et al., 2008), posterior callosal reductions were highly correlated with MMSE results and ICV, so that they failed to provide additional predictive power when these variables were included in the multi-factor regression. In contrast, reductions in anterior callosal area were associated with increased dementia symptoms independent of ICV and MMSE results, consistent with recent reviews highlighting the cognitive importance of the anterior callosum (Di Paola et al., 2010; Sexton et al., 2011). The same general conclusion from this OASIS mild dementia data set was independently drawn recently (Zhu et al., 2012) using a semi-automated callosal analyses and using only ICV as a covariate. Finally, image quality and segmentation performance did not appear to be limiting factors for the callosal measurement algorithm as applied to the older control and the patient populations that we analyzed.

## Summary

The C8 algorithm described in this study automatically isolates and measures callosum clusters from WM segmentations derived from structural MRI data sets. The procedures can provide reliable measurements of callosal area, regional thicknesses, and median length. The accuracy, reliability, robustness, and internal consistency were tested using two large public databases of images and used to analyze changes in the callosum due to normal aging and mild Alzheimer's dementia. Open-source software implementing the algorithm is available at www.nitrc.org/projects/c8c8.

## Disclaimer

The views expressed herein do not necessarily reflect the views of the US Department of Veterans Affairs or the United States Government.

### Conflict of interest statement

The authors declare that the research was conducted in the absence of any commercial or financial relationships that could be construed as a potential conflict of interest.

## Footnotes

^1^www.oasis-brains.org

^2^www.nitrc.org/projects/art

^3^fcon1000.projects.nitrc.org

^4^www.fil.ion.ucl.ac.uk/spm

^5^surfer.nmr.mgh.harvard.edu

^6^mirc.uzleuven.be/MedicalImageComputing/downloads/ems.php

^7^www.nitrc.org/projects/cleave

^8^www.mathworks.com

^9^www.r-project.org

^10^www.nitrc.org/projects/ccseg
